# CT differential diagnosis of COVID-19 and non-COVID-19 in symptomatic suspects: a practical scoring method

**DOI:** 10.1186/s12890-020-1170-6

**Published:** 2020-05-07

**Authors:** Lin Luo, Zhendong Luo, Yizhen Jia, Cuiping Zhou, Jianlong He, Jianxun Lyu, Xinping Shen

**Affiliations:** 1 Department of Radiology, The University of Hong Kong - Shenzhen Hospital, No.1, Haiyuan road Futian District, Shenzhen, 518000 China; 2 Department of Core Laboratory, The University of Hong Kong - Shenzhen Hospital, Hospital, No.1, Haiyuan road Futian District, Shenzhen, 518000 China

**Keywords:** Coronavirus infections, Pneumonia, Tomography, x-ray computed, Lung diseases

## Abstract

**Background:**

Although typical and atypical CT image findings of COVID-19 are reported in current studies, the CT image features of COVID-19 overlap with those of viral pneumonia and other respiratory diseases. Hence, it is difficult to make an exclusive diagnosis.

**Methods:**

Thirty confirmed cases of COVID-19 and forty-three cases of other aetiology or clinically confirmed non-COVID-19 in a general hospital were included. The clinical data including age, sex, exposure history, laboratory parameters and aetiological diagnosis of all patients were collected. Seven positive signs (posterior part/lower lobe predilection, bilateral involvement, rounded GGO, subpleural bandlike GGO, crazy-paving pattern, peripheral distribution, and GGO +/− consolidation) from significant COVID-19 CT image features and four negative signs (only one lobe involvement, only central distribution, tree-in-bud sign, and bronchial wall thickening) from other non-COVID-19 pneumonia were used. The scoring analysis of CT features was compared between the two groups (COVID-19 and non-COVID-19).

**Results:**

Older age, symptoms of diarrhoea, exposure history related to Wuhan, and a lower white blood cell and lymphocyte count were significantly suggestive of COVID-19 rather than non-COVID-19 (*p* < 0.05). The receiver operating characteristic (ROC) curve of the combined CT image features analysis revealed that the area under the curve (AUC) of the scoring system was 0.854. These cut-off values yielded a sensitivity of 56.67% and a specificity of 95.35% for a score > 4, a sensitivity of 100% and a specificity of 23.26% for a score > 0, and a sensitivity of 86.67% and a specificity of 67.44% for a score >  2.

**Conclusions:**

With a simple and practical scoring system based on CT imaging features, we can make a hierarchical diagnosis of COVID-19 and non-COVID-19 with different management suggestions.

## Background

The 2019 novel coronavirus disease (COVID-19) has become a global viral pandemic and a public health problem of international concern. According to the guidelines for COVID-19 (Trial Version 7th) China [1], confirmed COVID-19 cases need to be referred to a designated hospital while suspected cases need to be quarantined under medical surveillance. The medical care for quarantined patients and isolation for people with whom they have had close contact requires larger public health surveillance and response systems with an enormous medical burden. Chest CT can yield a quick positive result prior to positive real-time fluorescence polymerase chain reaction (RT-PCR), which is the gold standard for confirming COVID-19 at present [2] but with a notable false negative rate [3–5]. Although typical and atypical CT image findings are reported in several papers [2, 6–15], overlapping CT image features with viral pneumonia and other respiratory diseases also make an exclusion diagnosis difficult. We attempted to develop a simple and practical method to stratify cases requiring different repetition times of RT-PCR to identify highly suspicious cases and highly excluded cases.

## Methods

### Patients

We retrospectively enrolled 91 patients fulfilling the inclusion criteria: patients who underwent high-resolution CT within 7 days after the onset of symptoms and had the first consultation at the general hospital from Jan 10 to Feb 28, 2020. Of those 91 patients, 30 cases of COVID-19 were confirmed with WHO interim guidance, and 43 cases of other aetiology or clinically confirmed non-COVID-19 were finally included in our cohort. Suspected COVID-19 cases with abnormal chest CT findings (one COVID-19 and 1 non-COVID-19 with normal chest CT were excluded) were included with the following inclusion criteria [1]: (1) epidemiological exposure history within 14 days before the onset of symptoms – i) travel/residence history in Wuhan; ii) travel/residence history in Hubei but not Wuhan; iii) exposure history to confirmed cases or community, respiratory symptoms related patient; iv) cluster onset; (2) presented with fever and/or respiratory symptoms within 7 days of CT examination; and (3) normal or low white blood cell count and lymphocyte count at early onset. The exclusion criteria were as follows: (1) images with excessive motion artefact (one non-COVID-19 was excluded); (2) children and pregnant women (three COVID-19 and 9 non-COVID-19 were excluded); (3) lost to follow-up (three non-COVID-19).

The clinical data including age, sex, exposure history and laboratory parameters of all patients are summarized in Table 1

**Table 1:** The clinical data including age, sex, exposure history and laboratory parameters of all the collected patients

Parameter	All patients ***N*** = 73	RT-PCR confirmed COVID-19 ***N*** = 30	Laboratory or clinical confirmed not COVID-19 ***N*** = 43	Test statistic	***P*** value
Gender				0.329	0.566
Male	37 (50.7)	14 (46.7)	23 (53.5)		
Female	36 (49.3)	16 (53.3)	20 (46.5)		
Age(y)	41 (33–55.5)	54 (36–64)	37 (32–47)	3.091	0.002*
Exposure History				34.717	< 0.001*
Wuhan contact	29 (39.7)	24 (80.0)	5 (11.6)		
Hubei (not Wuhan) contact	10 (13.7)	2 (6.7)	8 (18.6)		
Not Hubei contact or Cluster onset	34 (46.6)	4 (13.3)	30 (69.8)		
Duration between CT and symptom onset (day)	3 (1–6)	3 (1–5.25)	3 (1–7)	−0.074	0.941
Symtoms					
Fever	45 (61.6)	22 (73.3)	23 (53.5)	2.943	0.086
Cough	49 (67.1)	17 (56.7)	32 (74.4)	2.523	0.112
Sputum production	19 (26.0)	5 (16.7)	14 (32.6)	2.318	0.128
Running/stuffy nose	3 (4.1)	0 (0.0)	3 (7.0)	2.183	0.264
Fatigue	8 (11.0)	2 (6.7)	6 (14.0)	1.016	0.314
Muscle ache/myalgia	15 (20.5)	7 (23.3)	8 (18.6)	0.242	0.623
Diarrhea	4 (5.5)	4 (13.3)	0 (0.0)	7.450	0.006*
Chest pain	1 (1.4)	0 (0.0)	1 (2.3)	1.068	0.301
Sore throat	10 (13.7)	2 (6.7)	8 (18.6)	2.307	0.129
Headache	8 (11.0)	3 (10.0)	5 (11.6)	0.048	0.826
Laboratory Investigation					
White-cell count(10^−9^ g/L) (normal range 3.89–9.93)	6.81 (5.40–8.37)	5.43 (4.25–6.12)	7.67 (6.73–9.06)	−4.547	< 0.001*
Neutrophil percentage(%) (normal range 44.0–72.0)	64.45 (57.70–73.45)	64.80 (61.35–73.38)	64.45 (55.38–73.70)	0.908	0.364
Neutrophil count(10^−9^ g/L) (normal range 2.01–7.42)	4.21 (3.30–5.96)	3.46 (3.00–4.32)	4.92 (3.75–7.09)	−3.295	0.001*
Lymphocyte percentage(%) (normal range 20.0–45.0)	26.15 (18.05–31.08)	25.25 (17.58–31.03)	26.75 (18.55–32.55)	−0.423	0.673
Lymphocyte count(10^−9^ g/L) (normal range 1.06–3.61)	1.70 (1.28–2.36)	1.32 (1.08–1.69)	1.93 (1.54–2.64)	−3.667	< 0.001*
Final diagnosis of non-COVID-19					
Influenza	2	–	2		
Mycoplasma pneumonia	7	–	7		
Pneumocystis carinii pneumonia	1	–	1		
Community-acquired pneumonia of unknown cause	33	–	33		

* *P* <0.05 was considered as statistical significant

Pathogenic evidence: a nucleic acid test by RT-PCR was used to detect the new coronavirus in respiratory samples. All enrolled patients had final diagnoses of twice-positive RT-PCR to confirm COVID-19, more than or equal to twice-negative RT-PCR (range 2–5 times) or at least one negative RT-PCR with other pathogens (mycoplasma pneumonia, human immunodeficiency virus and influenza) confirmed, or community-acquired pneumonia of unknown cause with resolved follow-up chest CT findings after treatment.

### CT image data acquisition

CT images of the thorax were acquired using the automatic exposure control setting and scan range, and the noise index of was 12.3. CT scans were performed ≤7 days after symptom onset on a helical 64-slice CT GE (Lightspeed Ultra 16, USA; 1.25 mm slice thickness; 1.5 pitch; 120 kVP tube voltage; 100–200 mAs tube current; sagittal and coronal reconstruction thickness, 3 mm with 3-mm intervals) or Siemens (Somatom Definition AS, Germany; 1 mm slice thickness; 1.2 pitch; 120 kVP tube voltage; 100–200 mAs tube current; sagittal and coronal reconstruction thickness, 3 mm with 3-mm intervals; and a sharp reconstruction kernel).

### CT image analysis

**Fig. 1:**
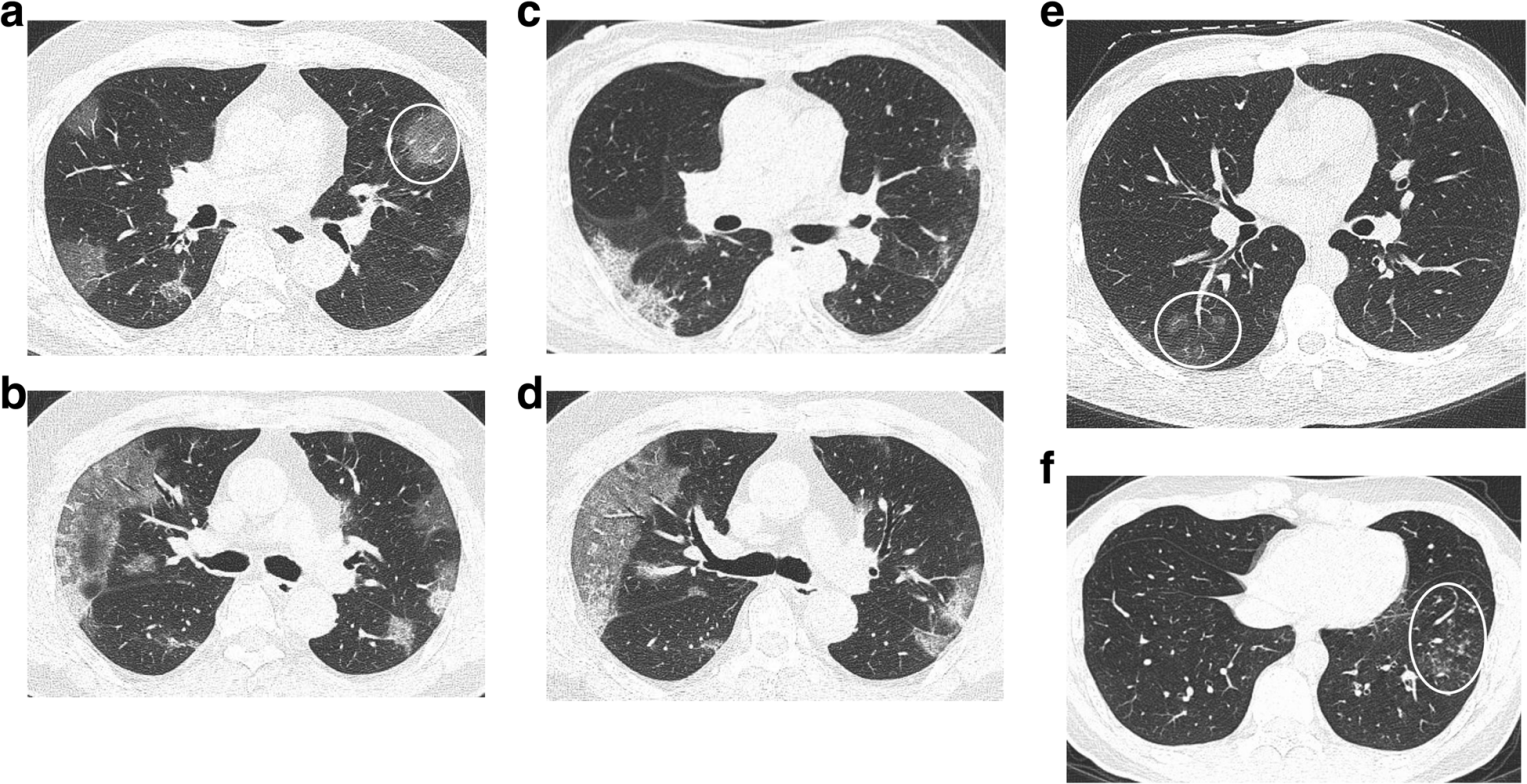
**a**-**d** are the images of COVID-19. a Pure GGO and Rounded GGO (circle). **b** Mix GGO and consolidation. **c** Crazy-paving pattern. **d** Subpleural bandlike areas of GGO. **e**-**f** are the images of non-COVID-19. **e** Central (peribronchovascular) distribution (circle). **f** Tree-in-bud sign (circle)

We summarized several significant COVID-19 CT image features by reviewing recently reported papers published or e-published on chest CT findings from the COVID-19 outbreak in China in Table 2. Referring to other CT image signs in viral pneumonia [16, 17] or community-acquired pneumonia [16, 18], we set seven positive signs from significant COVID-19 image features and four negative signs from significant image features of other non-COVID-19 pneumonia as in Table 3 and Fig. 1. In brief, visual scores were defined as follows: score 1, positive significant COVID-19 image features; score − 1, non-COVID-19 with viral pneumonia or community-acquired pneumonia of unknown cause image features. An overall score was reached by summing the scores of the eleven features in Table 4.

**Table 2:** significant COVID-19 CT image features by reviewing reported papers published or e-published of chest CT findings from COVID-19 outbreak in China recently

	GGO +/−consolidation	Peripheral/ subpleural distribution	Posterior part/ lower lobe predilection	Bilateral involvement	Crazy-paving pattern	Rounded GGO	> 2 lobes affected	Central distribution
**Kann et al[**6**]**	86%	33%	–	76%	19%	–	–	–
**Bernheim et al[**7**]**	91%	63%	–	73%	6%	65%	62%	–
**Chung et al[**8**]**	86%	33%	–	76%	19%	33%	71%	–
**Song et al[**9**]**	< 59%	86%	80%	86%	75%	–	91%	10%
**Pan F et al[**10**]**	75%	54%	–	42%	25%	–	58%	–
**Zu et al[**11**]**	(+++)	(+++)	(+++)	(+++)	(++)	–	–	–
**Pan YY et al[**12**]**	85.70%	–	–	–	–	–	69.7%	–
**Shi et al[**13**]**	65%	54%	–	79%	–	–	–	–
**Xu X et al[**14**]**	72%	51%	–	59%	12%	–	73%	–
**Xu YH et al[**15**]**	75%	< 96.4%	–	> 53.6%	75%	–	–	–
**Guan et al[**2**]**	56.40%	–	–	51.80%	–	–	–	–

*GGO* Ground glass opacification

**Table 3:** Selected chest CT image features and Scores analysis

	CT parameter	All patients N = 73	RT-PCR confirmed COVID-19 N = 30	Laboratory or clinical confirmed non-COVID-19 N = 43	χ^**2**^ statistic	***P***-value
**Positive + 1**	**Posterior part/lower lobe predilection**	66 (90.4)	30 (100.0)	36 (83.7)	7.923	0.005*
	**Bilateral involvement**	33 (45.2)	18 (60.0)	15 (34.9)	4.500	0.034*
	**Rounded GGO**	9(12.3)	8 (26.7)	1 (2.3)	10.226	0.001*
	**Subpleural bandlike GGO**	16 (21.9)	14 (46.7)	2 (4.7)	18.228	< 0.001*
	**Crazy-paving pattern**	37 (50.7)	23 (76.7)	14 (32.6)	13.755	< 0.001*
	**Peripheral distribution**	58 (79.5)	29 (96.7)	29 (67.4)	9.245	0.002*
	**GGO +/− consolidation**	66 (90.4)	27 (90.0)	39 (90.7)	0.010	0.921
**Negative − 1**	**Only one lobe involvement**	34 (46.6)	9 (30.0)	25 (58.1)	5.623	0.018*
	**Only Central distribution (peribronchovascular)**	15 (20.5)	1 (3.3)	14 (32.6)	9.245	0.002*
	**Tree-in-Bud sign (centrilobular nodules)**	6 (8.2)	0 (0.0)	6 (14.0)	6.723	0.010*
	**Bronchial wall thickening**	12 (16.4)	1 (3.3)	11 (25.6)	7.572	0.006*
**Total Score median (IQR)**		2 (1–4)	4 (2–5)	2 (0–2)	4.637	< 0.001

*GGO* Ground glass opacification

* *P* <0.05 was considered as statistical significant

**Table 4:** Discriminative performance of prediction for COVID-19

Variable	AUC	SE	*P*-value	95%CI
Combined CT Score	0.854	0.045	< 0.001*	0.752 to 0.926

* *P* <0.05 was considered as statistical significant

The image analysis focused on the features of each patient, including (a) number of lobes involved, (b) lesions and distribution characteristics (e.g., peripheral distribution, central distribution, subpleural distribution, and posterior distribution), (c) lesion patterns (e.g., ground glass opacification (GGO) with or without consolidation, crazy-paving pattern, and the shape of the GGO), (d) other signs in the lesion (e.g., bronchial and/or bronchiolar wall thickening), and (e) other findings (e.g., tree-in-bud sign). All CT findings were described according to the Fleischner Society recommendations and similar studies [19–21]. Peripheral distribution was defined as any lesion affecting a peripheral area (3–4 cm in thickness at the lung periphery) with or without central distribution. Central distribution was defined as ONLY central distribution (the central tubular structures in a secondary pulmonary lobule), and any lesion with a peripheral area affected was excluded. Ground glass opacification was defined as hazy opacity that did not obscure the underlying bronchial and vascular margins; consolidation was defined as opacification with obscuration of bronchial structures and pulmonary vessels [19](Fig. 1a, b). A crazy-paving pattern is ground-glass opacity superimposed with lines of reticular patterns [22](Fig. 1c). Rounded GGO is a round-shaped GGO in any plane (Fig. 1a). The subpleural bandlike GGO is a pronounced peripheral, subpleural distribution along with axial pleura (Fig. 1d). Central (peribronchovascular) distribution was defined as typically around the bronchiolar vascular bundle and sparing the subpleural surfaces. They are typically at least 5–10 mm away from the pleural surfaces [23] (Fig. 1e). The tree-in-bud sign was defined as peripheral, small, centrilobular, and well-defined nodules of soft-tissue attenuation connecting to linear, branching opacities that have more than one contiguous branching site [24] (Fig. 1f).

CT images were reviewed retrospectively and independently by two cardiothoracic radiologists (A with 25 years of experience and B with 15 years of experience) who knew that patients had suspected COVID-19 exposure history but were blinded to any other laboratory or RT-PCR data. When a discrepancy of image feature definition and diagnoses existed between the two radiologists, the final result was decided according to their consensus.

### Statistical analysis

Continuous variables were presented as medians with interquartile ranges (IQR). Categorical variables were summarized as counts and percentages. Differences between the two groups (confirmed COVID-19 vs. confirmed non-COVID-19) were compared for continuous and categorical variables by a Mann-Whitney U test and chi-squared test, respectively. *p* < 0.05 was considered significant. The receiver operating characteristic (ROC) curve was used to determine the cut-off value of COVID-19 prediction. The area under the curve (AUC) and Youden index were computed. The performance of each cut-off value was evaluated as sensitivity, specificity, positive and negative predictive values. All analyses were performed with MedCalc Statistical Software, version 18.11.3.

## Results

### Characteristics and clinical laboratory findings

This retrospective study included 73 patients, of which 30 were confirmed as COVID-19 positive by RT-PCR, and 43 were classified as non-COVID-19 who were finally confirmed by RT-PCR as COVID-19-negative and positive for other pathogens or clinical treatment (Table 1). In this study, 37 patients were male (50.7%) and 36 patients were female (49.3%). There was no significant difference in sex between these two groups; moreover, 53.3% of the COVID-19 group were male and 46.5% of the non-COVID-19 group were female. Patients in the COVID-19 group were significantly older (median age 54 years, IQR 36–64, *p* < 0.01) than those in the non-COVID-19 group (median age 37 years, IQR 32–47). The majority of the COVID-19 group had an exposure history related to Wuhan (*n* = 24, 80%) while most patients (*n* = 30, 69.8%) of non-COVID-19 group had no history of Hubei contact or cluster onset. Fever (61.6%) and cough (67.1%) were the most common symptoms presented in the majority of both groups. Only COVID-19 had 4 (13.3%) patients presenting with diarrhoea. In the non-COVID-19 group, 3 patients (7%) and 1 patient (2.3%) had symptoms of running/stuffy nose and chest pain, respectively, which were not present in the COVID-19 group in our study. The white blood cell count and lymphocyte count of all patients were within normal range. However, the white blood cell count of the COVID-19 group (median 5.43 g/L, IQR 4.25–6.12 g/L) was significantly lower (*p* < 0.001) than that of the non-COVID-19 group (median 7.67 g/L, IQR 6.73–9.06 g/L). The lymphocyte count in the COVID-19 group (median 1.32 g/L, IQR 1.08–1.69 g/L) was also significantly lower (*p* < 0.001) than that in the non-COVID-19 group (median 1.93 g/L, IQR 1.54–2.64 g/L).

### CT imaging findings

High-resolution chest CT scans were performed for all 30 patients with COVID-19 and 43 patients with non-COVID-19. Selected chest CT image features are shown in Table 3. Of the 7 positive signs, GGO with or without consolidation (*n* = 66, 90.4% in all patients) was one of the most common image features shared by the COVID-19 (*n* = 27, 90%) and non-COVID-19 (*n* = 39, 90.7%) groups, and it was not significantly different between the two groups (*p* = 0.921). Although other positive image features could be recognized in both the COVID-19 and non-COVID-19 groups, there were significant differences between the two groups (*p* < 0.05). In particular, rounded GGO (*n* = 8, 26.7%) and subpleural bandlike GGO (*n* = 14, 46.7%) were pretty common in COVID-19 but rarely seen in non-COVID-19 (rounded GGO, n = 1, 2.3%; subpleural bandlike GGO, *n* = 2, 4.7%, *p* < 0.001).

In the 4 negative signs, the tree-in-bud sign (centrilobular nodules) could only be detected in the non-COVID-19 group (*n* = 6.14%, *p* = 0.01) while other negative signs presented more in the non-COVID-19 group than in the COVID-19 group with a significant difference (*p* < 0.05).

**Fig. 2:**
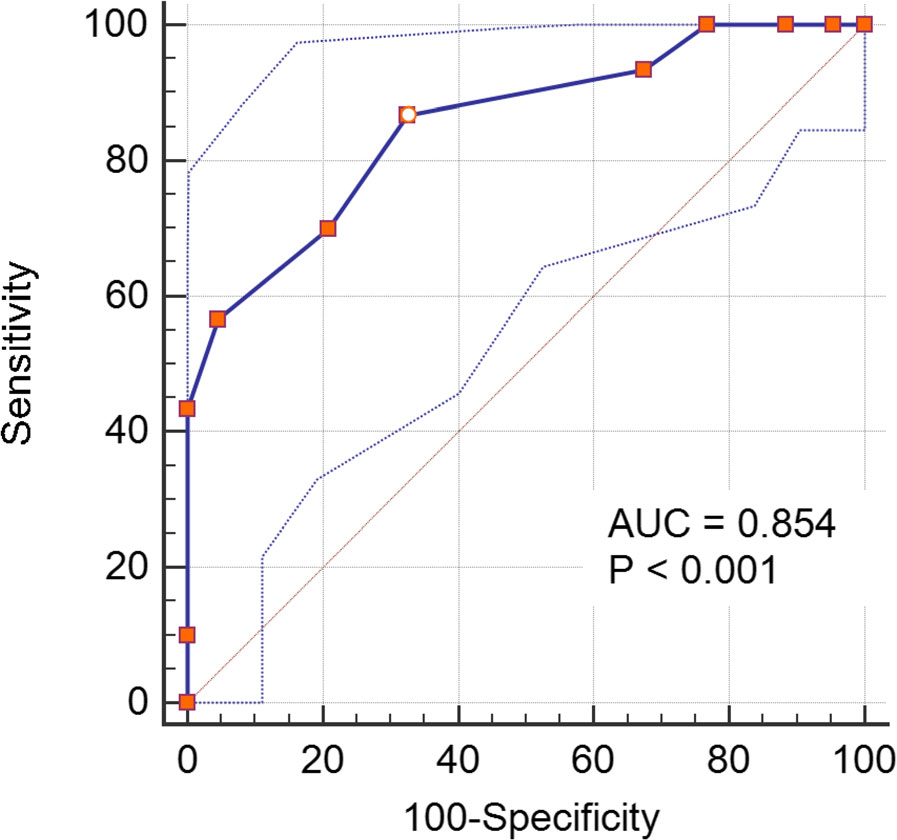
ROC curve for COVID-19 prediction

Based on the 11 CT image features listed in Table 3, we calculated the total score for each patient. The total score ranged from − 4 to 7. The median score of the COVID-19 group was 4 (IQR 2–5), which was significantly higher (*p* < 0.001) than that of the non-COVID-19 group (median 2, IQR 0–2). The performance of our scoring system for the diagnosis of COVID-19 is shown in Table 4. The receiver operating characteristic (ROC) curve of the combined CT image features analysis revealed that the area under the curve (AUC) of our scoring system was 0.854 (95%CI: 0.752–0.926), *p* < 0.001 (Fig. 2). The cut-off values yielded a sensitivity of 56.67% and a specificity of 95.35% for a score > 4, a sensitivity of 100% and a specificity of 23.26% for a score > 0, and a sensitivity of 86.67% and a specificity of 67.44% for a score >  2 (Table 5).

**Table 5:** Prediction performance of COVID-19 with different cut-off values

Cut-off	TP	FP	TN	FN	Youden Index	Sensitivity (95%CI)	Specificity (95%CI)	PPV (95%CI)	NPV (95%CI)
**> 4**	17	2	41	13	0.5202	56.67 (37.4 to 74.5)	95.35 (84.2 to 99.4)	89.5 (67.9 to 97.2)	75.9 (67.6–82.7)
**> 2**	26	14	29	4	0.5411	86.67 (69.3 to 96.2)	67.44 (51.5 to 80.9)	65.0 (54.2–74.5)	87.9 (74.0–94.9)
**> 0**	30	33	10	0	0.2326	100.0 (88.4 to 100.0)	23.26 (11.8 to 38.6)	47.6 (43.5–51.7)	100.0 (−)

## Discussion

COVID-19 is a severe and easily transmissible infectious disease spreading all around the world. Chest CT examination plays a vital role in the initial and early diagnosis of COVID-19 [8]. Positive chest CT can be obtained before the initial positive RT-PCR. Given the varied isolation and treatment principles of suspected COVID-19 with epidemic history, it is important to focus on baseline CT findings that radiologists first encounter to differentiate non-COVID-19 from COVID-19 in the patients’ first consultation at a general hospital. Although Bai et al. [25] revealed that radiologists were capable of distinguishing COVID-19 from viral pneumonia upon chest CT with high specificity and moderate and varying specificity (24–94%) among 7 different readers from China and the USA, an easily understood and simple method is still urgently needed in epidemic areas, especially areas lacking medical resources and well-trained radiologists. Compared with non-COVID-19 patients, COVID-19 patients are more likely to present with some CT image features according to previous studies. We have summarized the 7 most common imaging features in COVID-19 patients as a positive score point. All of the positive score points assessed in our study were significantly different between the two groups, except for GGO with or without consolidation. GGO can result from the pathology of alveolar damage filling with blood, pus, water or cells [9, 26] in viral infections, including COVID-19, and bacterial infections. GGO with or without consolidation is to some extent related to the different course of the disease. However, consolidation would be increased in the progressive stage (5–8 days) [10]. To reduce interference by consolidation, our study confined the cohort to within 7 days after onset of symptoms. The small COVID-19 virus, 60–140 nm in diameter [1], could go straight to the terminal alveoli, reasonably favouring peripheral distribution while other, much larger pathogens would not pass through the alveolar pores easily. The ‘crazy-paving’ pattern results from thickening of the interlobular septa, and it can be seen primarily in any airspace, interstitial, or mixed disease [27]. Rounded GGO as well as subpleural bandlike GGO are very conspicuous and characteristic signs at the first glance on examination of the COVID-19 CT images. Although there was no explanation for these two signs, they could be easily detected; we used them as our positive scoring points.

In our design, we added some negative points to make a hierarchical diagnosis. Based on the fact that most of the reported COVID-19 cases affected more than 2 lobes of the lungs, only one lobe involvement was taken as a negative scoring point. Meanwhile, single-lobe infection has been reported in some cases of community-acquired pneumonia [28]. Some progressive COVID-19 cases may affect the central area from the peripheral lung [15]. An image showing only central (peribronchovascular) distribution indicates distal small airway wall destruction or peribronchovacular infection, which is similar to the reason for the tree-in-bud pattern. The pathogenesis of bronchial wall thickening can be inflammatory damage of the bronchial wall, which may serve as a potential indicator for bacterial pneumonia [29], resulting in the destruction of bronchial wall structure and proliferation of fibrous tissue fibrosis [19]. It is more likely to present in severe COVID-19 patients but rarely in ordinary patients at the early stage. All three signs strongly indicated non-COVID-19 infection and were taken as distinctive negative scoring points.

Based on these typical image features of COVID-19 and other common non-COVID-19 pneumonia, a simple and practical scoring system has been established in our study. When tested in our group of suspected COVID-19 cases, the scoring system achieved good diagnosis performance with AUC = 0.854 (95%CI: 0.752–0.926). Our larger cohorts and good-to-excellent diagnostic performance confirmed a similar study of Himoto et al. [30] in Japan with simpler criteria and moderate-to-excellent inter-reader concordance. Their study proposed a statistically proven powerful tool for triaging patients based on positive COVID-19 image features while ours added some negative values. Using a score > 4 as a cut-off, our scoring system showed a high specificity of 95.35% (95%CI: 84.2 to 99.4%) and made only 2 false-positive diagnoses (false-positive rate: 4.65%); a score > 4 could be strongly suspected for COVID-19. For suspected cases with a score > 4, even negative results were shown several times by RT-PCR; hence, we still suggest that repeat RT-PCR testing is necessary. If using a score > 0 as the diagnostic cut-off, the sensitivity is 100% with no false-negative (0%) diagnoses of COVID-19; thus cases with scores ≤0 are less likely to be COVID-19. We can exclude COVID-19 in these cases with more confidence and reduce the testing by RT-PCR. Patients with scores of 0–4 should be classified as suspected COVID-19 and be quarantined under medical surveillance followed by at least two RT-PCR tests according to the suggestion of the newest edition of guidelines.

There are several limitations in our study. First, the analysis in our study was limited to one general hospital, but all cases had fulfilled the suspected COVID-19 criteria with exposure history, respiratory symptoms and normal or decreased white blood cell and lymphocyte counts. Our criteria might have missed rare cases with normal chest CT and negative RT-PCR at the first consultation. Second, with a small number of confirmed cases, we cannot set another group to verify the reliability of the scoring system. Future studies including more confirmed patients and multicentre studies would optimize the practical applicability of the scoring system and enable the verification of its reliability.

## Conclusion

With exposure history and respiratory symptoms in this epidemic period, the present simple scoring system provides rapid detection, which may enable better control of COVID-19 spread through medical management as well as reduce the larger public health surveillance and response systems.

## Data Availability

The datasets analyzed during the current study available from the corresponding author on reasonable request.

## Abbreviations

COVID-19: 2019 novel coronavirus disease
RT-PCR: Reverse transcription-polymerase chain-reaction
GGO: Ground glass opacification
